# Identification of *EGFR*, *MMP9*, and *AGTR1* as actionable therapeutic targets in acetyl tributyl citrate-driven breast cancer: integrated network toxicology and *in vitro* validation

**DOI:** 10.3389/fphar.2026.1896118

**Published:** 2026-08-25

**Authors:** Bo Zhou, Lisi Zhou, Yue Zhang, Xiangyang An

**Affiliations:** 1 Taishan Academy of Medical Sciences, The Affiliated Taian City Central Hospital of Qingdao University, Taian, Shandong, China; 2 Department of Esophageal and Gastrointestinal Oncology, The Affiliated Taian City Central Hospital of Qingdao University, Taian, Shandong, China; 3 Outpatient Department, The Affiliated Taian City Central Hospital of Qingdao University, Taian, Shandong, China; 4 Department of General Obstetrics, The Affiliated Taian City Central Hospital of Qingdao University, Taian, Shandong, China

**Keywords:** acetyl tributyl citrate, bioinformatics, breast cancer, molecular docking, network toxicology

## Abstract

**Background:**

Growing exposure to acetyl tributyl citrate (ATBC) drives breast cancer (BC) progression and poses significant challenges to clinical management. However, the precise molecular targets that mediate ATBC-induced tumor progression remain largely elusive. Identifying these cell surface and intracellular targets is critical for the development of rational, integrated targeted therapeutic strategies.

**Methods:**

A comprehensive Network Toxicology approach combined with multi-omics bioinformatics was employed to elucidate the molecular targets of ATBC in BC. Core hub genes were identified by intersecting potential ATBC targets with BC-related differentially expressed genes (DEGs). The diagnostic and immunological significance of these targets was evaluated. Finally, molecular docking and *in vitro* functional assays on BC cell lines were performed to validate target engagement and therapeutic potential.

**Results:**

ATBC exposure demonstrated potential carcinogenic risk and promoted BC progression. We identified three critical hub targets: *EGFR*, *MMP9*, and *AGTR1*. Molecular docking confirmed high binding affinities between ATBC and these target proteins. *In vitro* validation revealed that ATBC significantly promoted cell proliferation and migration, and suppressed apoptosis in ER-positive MCF-7 cells, a mechanism driven by profound upregulation of *EGFR*, *MMP9*, and *AGTR1* mRNA expression. Furthermore, the expression of these targets correlated significantly with immune cell infiltration within the tumor microenvironment.

**Conclusion:**

ATBC may promote BC progression through upregulation of *EGFR*, *MMP9*, and *AGTR1*. These genes represent both potential diagnostic biomarkers and actionable therapeutic targets, providing a rational basis for repurposing existing *EGFR* inhibitors and angiotensin receptor blockers to treat environmentally driven BC.

## 1 Introduction

Breast cancer (BC) ranks among the most prevalent malignant neoplasms endangering the survival and wellbeing of women worldwide, with its incidence and mortality rates consistently listed among the top for all malignancies affecting the female population (Global, 2026). Despite significant progress in the early screening, diagnosis, and treatment of BC in recent years, its etiology remains highly complex and is modulated by a multitude of contributing factors (Hickey et al., 2024; Conner et al., 2024). Of note, a growing body of evidence has confirmed that environmental exposure exerts a pivotal role in the tumorigenesis and malignant progression of BC (Li J. et al., 2025).

Acetyl tributyl citrate (ATBC) is a widely used phthalate-alternative plasticizer characterized by stable chemical properties and good biodegradability. It has long been considered a safe alternative to toxic phthalate plasticizers and is widely utilized across a diverse range of fields, including PVC materials, cosmetics, adhesives, and medical devices (Zheng et al., 2025; Zhang D. et al., 2024). ATBC is considered a safe plasticizer and only becomes toxic at extremely high exposure concentrations (Lin et al., 2025). However, ATBC can readily be released and migrate from consumer products, entering a wide range of environmental matrices and biological media, including air, water, soil, food, and blood (Huang, 2023). The general population is exposed to ATBC via multiple routes, such as dermal contact, ingestion of contaminated food and pharmaceuticals, and inhalation of indoor dust (Zhang et al., 2023; Takeshita et al., 2011). With the expanding application of ATBC, this compound has been frequently detected at high concentrations in diverse environmental and biological matrices, raising serious concerns over its potential long-term human exposure risks and carcinogenic potential. Using toxicological predictions, we found that ATBC exhibits potential carcinogenicity. Nevertheless, the specific roles and underlying molecular mechanisms linking ATBC exposure to the initiation and progression of BC remain largely unknown.

Despite these concerns, from the perspective of tumor pharmacology and precision medicine, it remains largely unknown how such environmental pollutants reshape the signaling networks of tumor cells. Specifically, the exact mechanisms by which environmental factors bind to or aberrantly activate specific cell surface receptors and intracellular targets have not been fully elucidated. Identifying these environment-driven oncogenic targets is of urgent clinical significance for developing and optimizing integrated targeted therapies and precision medicine strategies for BC.

Network Toxicology and systems biology integrate bioinformatics, multi-omics data, and computational modeling to comprehensively analyze complex biological interactions and drug-target networks. Combined with molecular docking, this approach enables the verification of binding affinities and interaction mechanisms between exogenous compounds and critical biomolecules (Zhang et al., 2025; Cui et al., 2025; Li Y. et al., 2025). This integrated research model provides an innovative strategy not only for investigating the molecular mechanisms of environmental pollutants but also for discovering novel therapeutic targets.

This study aims to systematically elucidate the core molecular targets mediating the progression of BC driven by ATBC exposure using an integrated Network Toxicology and multi-omics approach. We hypothesize that ATBC may act as an exogenous ligand or signaling modulator, aberrantly activating specific cell membrane receptors and intracellular key kinases. By employing this multidimensional framework and *in vitro* cell experiments, we seek not only to uncover the pro-tumorigenic mechanisms of ATBC but, more importantly, to identify highly actionable therapeutic targets. Ultimately, our findings aim to provide a robust scientific basis for drug repurposing and the development of integrated targeted therapies to combat environmentally driven BC progression.

## 2 Materials and methods

### 2.1 Network toxicology and multi-omics bioinformatics analysis

#### 2.1.1 Toxicological profiling of ATBC

To comprehensively evaluate the properties and safety profile of ATBC, computational predictions were conducted using the ProTox-3.0 (https://tox.charite.de/protox3/) and ADMETlab 3.0 (https://admetlab3.scbdd.com/) platforms. These *in silico* analyses provided a macroscopic assessment of the ADMET (Absorption, Distribution, Metabolism, Excretion, and Toxicity) characteristics and potential hazards associated with ATBC exposure.

#### 2.1.2 Prediction of potential targets of ATBC

The SMILES sequence of ATBC was imported into the SwissTargetPrediction web server (http://www.swisstargetprediction.ch/) for the prediction of its potential binding targets, restricting the species to *Homo sapiens*. Concurrently, experimentally validated bioactive targets were retrieved from the ChEMBL database (http://www.ebi.ac.uk/chembl/) using its standard compound name, filtering for human-derived targets. All retrieved targets were uniformly annotated and converted to the official human gene symbols using the UniProt Knowledgebase (https://www.uniprot.org/). Target sets from both databases were merged and deduplicated to generate a comprehensive list of potential ATBC targets.

#### 2.1.3 Acquisition of BC-associated targets

BC-associated disease targets were systematically retrieved from three authoritative human gene databases, including GeneCards (https://www.genecards.org/), DisGeNET (https://www.disgenet.org/), and OMIM (https://omim.org/), with “breast cancer” as the core search term. The targets were subsequently merged and deduplicated to construct a non-redundant BC disease target library.

#### 2.1.4 Identification of intersection targets and protein-protein interaction (PPI) network construction

VENNY 2.1 was adopted to identify the overlapping targets between ATBC potential targets and BC-related targets, representing the key targets of ATBC in BC. These overlapping targets were then submitted to the STRING database to construct a PPI network with a high-confidence interaction cutoff of >0.7. The network was visualized using Cytoscape 3.10.0, and core topological parameters (Degree, Betweenness Centrality, and Closeness Centrality) were calculated via the CytoNCA plugin. The top 20 targets ranked by Degree were designated as hub targets.

#### 2.1.5 Functional and pathway enrichment analysis

We performed functional and pathway enrichment analysis on the intersecting targets using the integrated online bioinformatics platform (https://www.bioinformatics.com.cn/). Gene Ontology (GO) enrichment analysis was conducted across three functional domains: Biological Process (BP), Cellular Component (CC), and Molecular Function (MF). In parallel, Kyoto Encyclopedia of Genes and Genomes (KEGG) pathway enrichment analysis was carried out. GO terms and KEGG pathways with an adjusted *P* < 0.05 were considered statistically significant, and the results were visualized as bar charts and bubble plots, respectively.

#### 2.1.6 Differentially expressed genes (DEGs) and core target screening

We obtained data from the NCBI Gene Expression Omnibus database (GEO, https://www.ncbi.nlm.nih.gov/geo/). Retrieve and download the BC mRNA expression profile dataset that meets the MIAME standard. All data are stored in the standardized MINIML format. Based on the R statistical analysis environment (version 4.5.2), a linear model was constructed using the limma package for gene expression differential analysis. The screening criteria for DEGs were set as a corrected *P* < 0.05 and | log _2_ (fold change) |>4. Further utilize the clusterProfiler package to perform GO functional enrichment analysis and KEGG pathway enrichment analysis on the selected DEGs. Finally, the top 20 key targets in the aforementioned PPI network sorted by degree value are cross mapped with BC DEGs, so as to identify the core treatable hub gene that drives BC in this study.

#### 2.1.7 Clinical validation and immune infiltration analysis

To evaluate the clinical and therapeutic relevance of the hub genes, their expression levels across different pathological stages and their correlations with immune cell infiltration profiles within the tumor microenvironment were analyzed. The diagnostic predictive performance was evaluated by constructing receiver operating characteristic (ROC) curves, with diagnostic efficacy stratified according to the area under the curve (AUC) values as follows: poor efficacy (AUC ≤ 0.5), moderate efficacy (0.5 < AUC < 0.7), and excellent efficacy (AUC > 0.7).

#### 2.1.8 Subcellular localization of targets and its therapeutic significance

To align with targeted therapy strategies against specific cellular compartments, the Human Protein Atlas (THPA, https://www.proteinatlas.org/) was utilized to determine the subcellular localization and expression distribution of the core genes in BC tissues. The subcellular localization of the target directly determines drug delivery strategies and treatment plans. This positioning feature can provide theoretical basis for target screening and subsequent *in vitro* validation.

#### 2.1.9 Molecular docking and visualization analysis

Download the ligands SDF file from the PubChem database and convert it to MOL2 format using Open Babel GUI. The PDB Format structure file of the target protein was obtained from the Protein Data Bank (PDB) database. Molecular docking was carried out using AutoDock Tools 1.5.7 and AutoDock Vina, and the final docking results were visualized with PyMOL 2.4.

### 2.2 *In vitro* pharmacological validation

#### 2.2.1 Cell lines and reagent preparation

Human BC cell lines MCF-7 (estrogen receptor positive) and MDA-MB-231 (triple negative) were routinely subcultured in Dulbecco’s Modified Eagle Medium (DMEM) containing 10% fetal bovine serum (FBS) and 1% penicillin streptomycin double antibody mixture. All cells were placed in a constant temperature incubator at 37 °C, and 5% CO_2_ saturation humidity for standard sterile culture, and only logarithmic growth phase cells were taken for subsequent ATBC exposure experiments.

ATBC powder was fully dissolved in dimethyl sulfoxide (DMSO) to prepare a high concentration mother liquor. After filtration and sterilization through a 0.22 μm sterile filter membrane, it was packaged and frozen at −20 °C in the dark. Before use, it was diluted with serum-free DMEM medium to the predetermined working concentration. To eliminate the interference of solvents on the biological behavior of cells, the final volume fraction of DMSO in the culture medium of all experimental and control groups was strictly controlled below 0.1%.

#### 2.2.2 Basic principles of ATBC concentration selection

This study selected ATBC concentrations of 100–1,600 μmol/L based on the following criteria (Global, 2026): Previous *in vitro* toxicology studies have confirmed that ATBC exhibits biological activity at micromolar concentrations (Zhang D. et al., 2024; Wu et al., 2025); (Hickey et al., 2024) Our preliminary experiment showed that <100 μmol/L had a weak effect on BC cell proliferation within 24 h, while >1,600 μmol/L induced non-specific toxicity (Conner et al., 2024); Although human exposure levels are mostly at the nanomolar level, ATBC can accumulate in tissues such as fat, and local concentrations may be higher than blood levels; And *in vitro* experiments require higher concentrations to induce acute effects in a short period of time (Li J. et al., 2025). ATBC has weak estrogenic and endocrine disrupting activity (Zhang D. et al., 2024). This study is a preliminary exploration of the mechanism, and the related effects still need to be further validated using physiological dosage and long-term exposure models.

#### 2.2.3 Cell viability assay (CCK-8)

Take MCF-7 and MDA-MB-231 cells in logarithmic growth phase, digest them with 0.25% trypsin EDTA to prepare single-cell suspension, adjust the cell concentration to 1 × 10^5^ cells/mL, and inoculate them into a 96 well cell culture plate at a volume of 100 μL per well, with 1 × 10^4^ cells per well. Place the culture plate in a constant temperature incubator at 37 °C and 5% CO_2_ saturation humidity for 24 h. When the cells are completely attached to the wall and the confluence degree reaches 30%–40%, discard the original culture medium in the well. Six ATBC treatment groups were set up in the experiment, with final concentrations of 0, 100, 200, 400, 800, and 1,600 μmol/L, respectively. At the same time, a solvent control group containing equal volumes of DMSO was set up to eliminate the non-specific effects of the solvent itself on cell activity. Each treatment group is equipped with 3 parallel wells, and all experiments are independently repeated at least 3 times to ensure the reproducibility and statistical reliability of the experimental results.

After 24 h of drug intervention, carefully add 10 μL of CCK-8 detection reagent to each well. During the operation, avoid generating bubbles to interfere with the detection results. Gently shake the culture plate horizontally to fully mix the reagent with the culture medium, and then continue to incubate in a 37 °C incubator in the dark for 60 min. Immediately after incubation, use an enzyme-linked immunosorbent assay (ELISA) reader to measure the absorbance values (OD _450_) of each well at a wavelength of 450 nm, with 600 nm as the reference wavelength, after deducting background interference from the culture plate and medium. All CCK-8 experiments were performed in triplicate.

#### 2.2.4 Apoptosis analysis via flow cytometry

Take MCF-7 and MDA-MB-231 cells in logarithmic growth phase, prepare a single-cell suspension by digestion with 0.25% trypsin EDTA, adjust the cell concentration to 1 × 10^5^ cells/mL, and inoculate them into a 12 well cell culture plate at a volume of 1 mL per well, that is, inoculate 1 × 10^5^ cells per well. Place the culture plate in a 37 °C, 5% CO_2_ saturated humidity incubator for routine cultivation. When the cell confluence reaches 70%–80%, discard the original culture medium and add fresh DMEM medium a containing gradient concentration of ATBC and an equal volume of DMSO solvent control. Strictly control the final concentration of DMSO in each group to be below 0.1% (v/v) to eliminate the cytotoxic effect of the solvent itself.

After 24 h of drug action, collect all cells: first, collect the culture supernatant containing suspended apoptotic/necrotic cells in the well, then gently digest the adherent cells with EDTA free trypsin, terminate digestion, and combine the cell suspension with the previously collected supernatant. Centrifuge at 4 °C and 1,000 rpm for 5 min and discard the supernatant. Gently resuspend the cells in pre cooled phosphate buffered saline (PBS) and wash twice. Subsequently, double staining was performed strictly in accordance with the instructions of the Annexin V-FITC/7-AAD cell apoptosis detection kit. The staining process was carried out in the dark and incubated at room temperature for 15 min. After incubation, immediately filter out cell clusters using a 40 μm cell sieve, and detect apoptotic cell subpopulations using Aurora full spectrum flow cytometry. Set a time gate for high-speed collection for 60 s, and collect more than 1 × 10^4^ cells per sample. Finally, FlowJo V10 software was used to perform gate analysis on the raw data and quantitatively calculate the apoptosis rate. All experiments were independently repeated three times. All flow cytometry experiments were performed in triplicate.

#### 2.2.5 Wound healing migration assay

Take MCF-7 and MDA-MB-231 cells in logarithmic growth phase, digest them with 0.25% trypsin EDTA to prepare a single-cell suspension, adjust the cell concentration to 5 × 10^4^ cells/mL, and inoculate them into a 12 well cell culture plate at a volume of 2 mL per well. Place the culture plate in a 37 °C, 5% CO_2_ saturated humidity incubator for routine cultivation. When the cells grow to 90% confluence, use a marker pen to draw three parallel horizontal marking lines at the bottom of the well plate (with an interval of about 3 mm between each well) as the positioning reference for subsequent photography. Subsequently, use a sterile 200 μL pipette tip perpendicular to the bottom marking line to draw uniform straight scratches on the cell monolayer, with 3 parallel scratches per well to ensure the reproducibility of the results.

Gently wash the cells three times with pre warmed serum-free DMEM medium to thoroughly remove any scratched cell debris and unattached cells. Then, add serum-free DMEM medium containing a gradient concentration of ATBC and an equal volume of DMSO solvent control. Strictly control the final concentration of DMSO in each group to be below 0.1% (v/v) to eliminate solvent interference. Transfer the culture plate to the Incucyte S3 live cell imaging analysis system and perform continuous dynamic monitoring under standard culture conditions. Automatically collect bright field images every 2 h and monitor continuously for 48 h, with a focus on recording scratch area images at 0 h (immediately after scratching) and 48 h. Quantitative analysis of images was performed using ImageJ 1.8.0 software: the average width of scratches at the same marked position was measured at 0 h and 48 h, respectively, and the cell migration rate was calculated. All Wound Healing experiments were performed in triplicate.

#### 2.2.6 Quantitative real-time PCR

Following ATBC exposure, total cellular RNA was isolated from MCF-7 and MDA-MB-231 BC cells using the TRIzol reagent in strict accordance with the manufacturer’s standard operating procedure. The extracted RNA was then reverse transcribed into complementary DNA (cDNA) for subsequent transcriptional analysis. Quantitative real-time polymerase chain reaction (qRT-PCR) was performed using the KAPA SYBR Green Fast kit on a Bio-Rad CFX96 real-time fluorescence detection system. The relative mRNA expression levels of *EGFR*, *MMP9*, and *AGTR1* were calculated using the 2^−ΔΔCt^ method, with β-actin serving as the stable endogenous reference gene for normalization. Primer sequences are shown in Table 1.

**TABLE 1:** qRT-PCR Primer Sequence Information.

Gene	Primer type	Primer sequence (5′–3′)	GC content (%)	TM value
*EGFR*	Forward primer	ATCATACGGGGAGGACCA	58	59.23
*EGFR*	Reverse primer	TCT​GAC​CGG​AGT​CCC​CAA​C	60	58.95
*MMP9*	Forward primer	ACG​CAC​GAC​GTT​TCC​AGT​A	55	58.41
*MMP9*	Reverse primer	CCA​CCT​GGT​TCA​ACT​CAC​TCC	57	57.90
*AGTR1*	Forward primer	ACC​TGT​ACG​CTA​GTG​TGT​TTC​TAC	46	56.33
*AGTR1*	Reverse primer	GCCAGCAGCCAAATGATG	52	57.05

qRT-PCR, real-time fluorescence quantitative polymerase chain reaction; GC, guanine cytosine content; TM, chain melting temperature.

### 2.3 Statistical analysis

All statistical analyses of experimental data were conducted using GraphPad Prism 8.0 software. Quantitative results are expressed as the mean ± standard deviation (SD) derived from at least three independent biological replicates. One-way analysis of variance (ANOVA) was used for statistical comparisons across multiple experimental groups, whereas an independent Student’s t-test was applied for pairwise comparisons between two groups. *P* < 0.05 was considered to represent a statistically significant difference.

## 3 Results

### 3.1 *In silico* toxicity and safety profiling of ATBC

**FIGURE 1:**
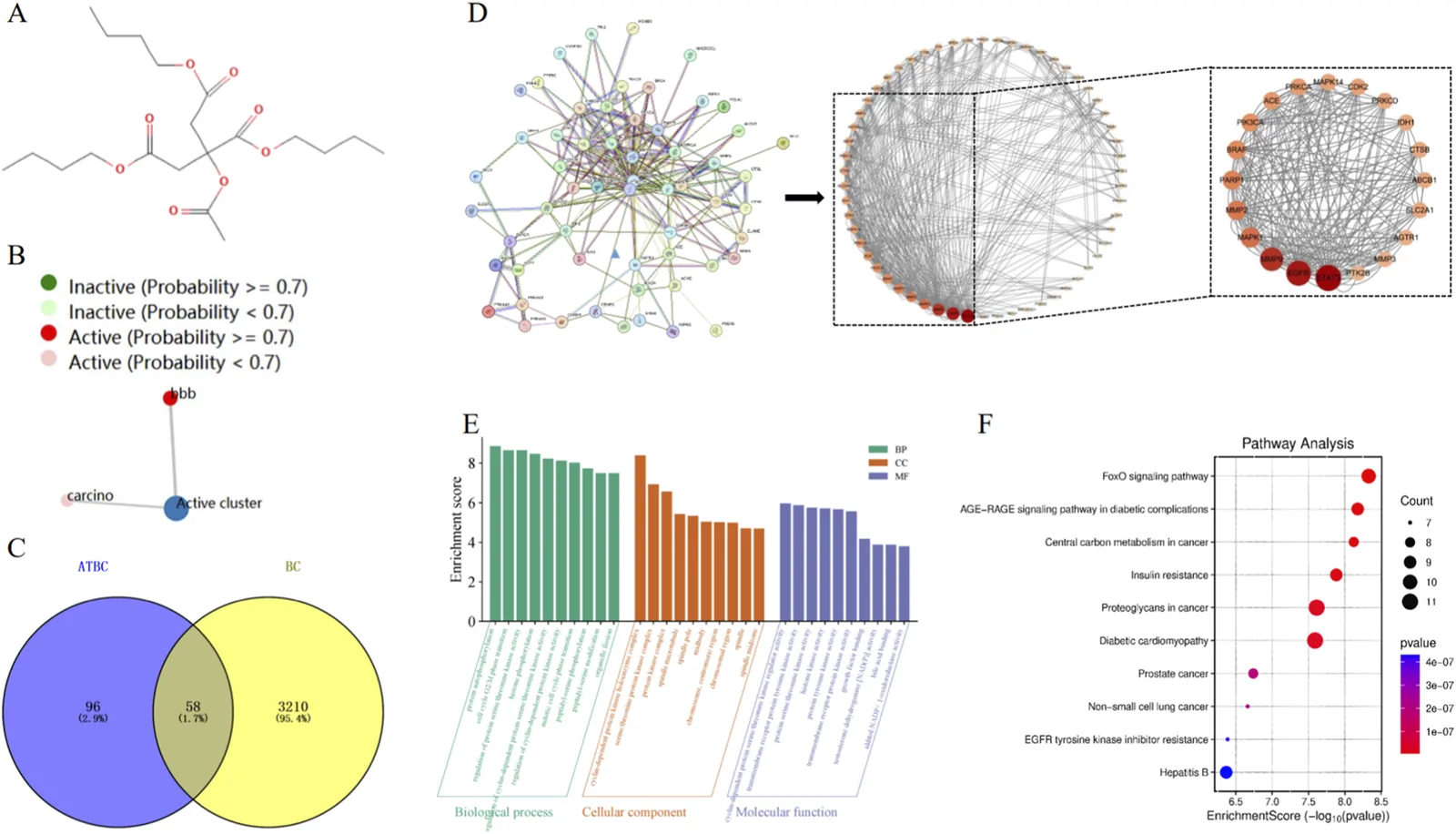
Network Toxicological Analysis of ATBC on BC. **(A)** ATBC molecular structure; **(B)** ATBC toxicity prediction results; **(C)** ATBC and BC target Venn diagram; **(D)** ATBC-BC intersection target PPI network and core targets; **(E)** GO function enrichment analysis; **(F)** KEGG pathway enrichment analysis.

Toxicity prediction of ATBC was conducted using the ProTox-3.0 and ADMETlab 3.0 databases. The predictive profiling showed that the potential toxicity of ATBC primarily involved hERG Blockers (10um), Hepatotoxicity (DILI), Skin Sensitization, Eye Irritation, Carcinogenicity, and BBB-barrier (BBB), and other aspects (Figures 1A,B; Table 2).

**TABLE 2:** Toxicity prediction results of ATBC.

Database	Property	Probability
ProTox-3.0	Carcinogenicity	0.64
ProTox-3.0	BBB-barrier (BBB)	0.92
ADMETlab 3.0	hERG blockers (10um)	0.53
ADMETlab 3.0	Hepatotoxicity (DILI)	0.39
ADMETlab 3.0	Skin sensitization	0.40
ADMETlab 3.0	Eye irritation	0.59

### 3.2 Systems pharmacology network construction and functional enrichment

Following screening and deduplication, 154 potential targets of ATBC and 3,268 BC-related disease targets were identified. Intersection analysis using VENNY 2.1 yielded a total of 58 overlapping targets between ATBC and BC (Figure 1C). A PPI network of these overlapping targets was constructed, consisting of 58 nodes and 230 edges. The network had an average node degree of 7.93, an average clustering coefficient of 0.617, and a PPI enrichment *P* < 1.0e-16. The top 20 hub targets ranked by degree included *STAT3*, *EGFR*, *MMP9*, *MAPK1*, *MMP2*, *PARP1*, *BRAF*, *PIK3CA*, *ACE*, *PRKCA*, *CDK2*, *MAPK14*, *PRKCD*, *CTSB*, *IDH1*, *SLC2A1*, *ABCB1*, *MMP3*, *AGTR1*, and *PTK2B* (Figure 1D).

GO functional enrichment analysis revealed 2,443 BP terms, 183 CC terms, and 281 MF terms. The BP terms mainly involved cellular response to chemical stress, response to oxidative stress, peptidyl-serine modification, and peptidyl-serine phosphorylation. The CC terms mainly involved the apical plasma membrane, vacuolar lumen, and endolysosome. The MF terms mainly involved amide binding, endopeptidase activity, and protein serine kinase activity (Figure 1E). KEGG pathway enrichment analysis identified 236 significantly enriched pathways. These pathways were primarily associated with the FoxO signaling pathway, central carbon metabolism in cancer, and proteoglycans in cancer (Figure 1F).

### 3.3 Multi-omics identification and clinical validation of hub targets

**FIGURE 2:**
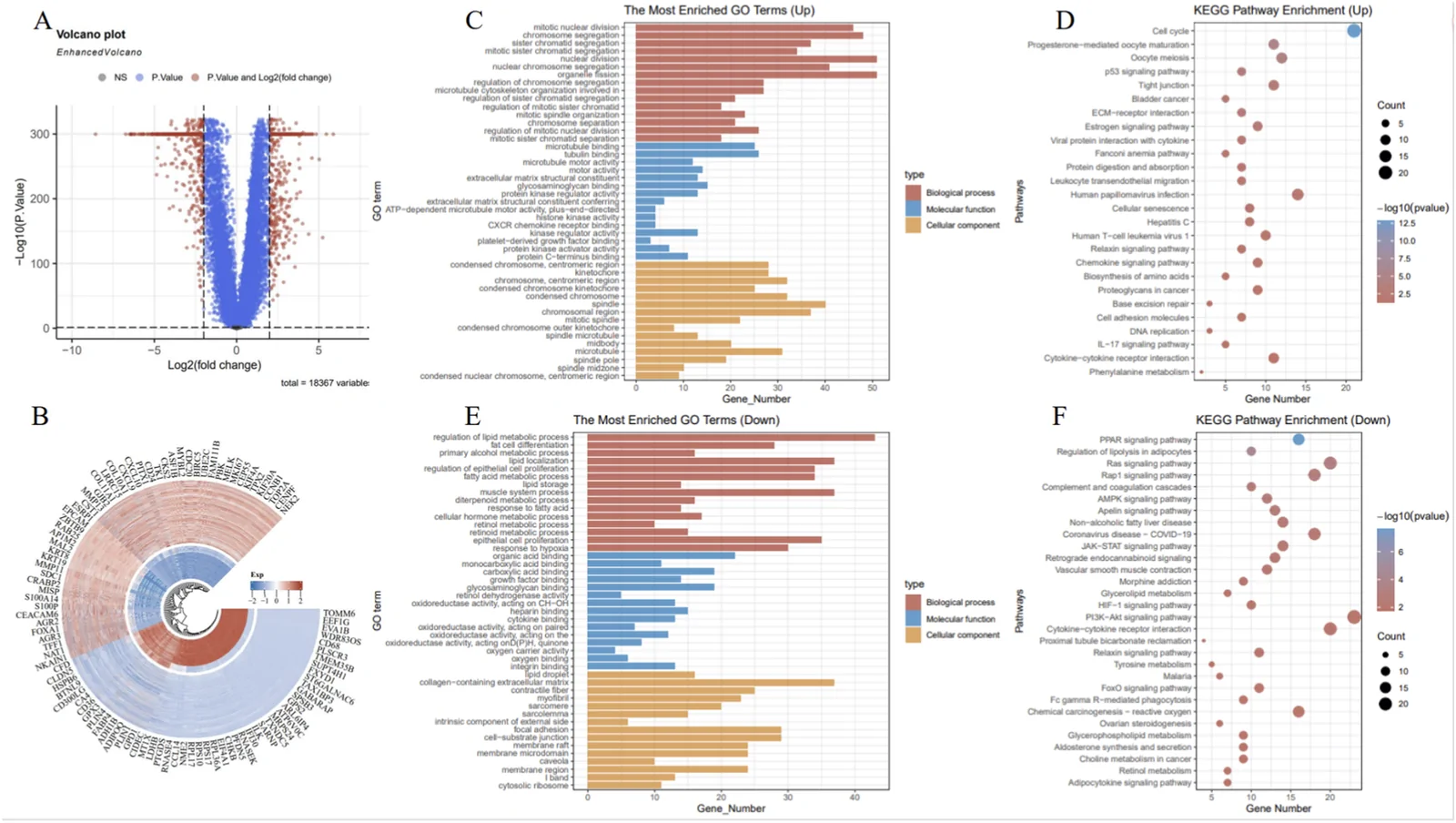
GO and KEGG Enrichment Analysis of DEGs in BC. **(A)** Volcanic map of DEGs; **(B)** Differential expression gene heatmap; **(C)** GO functional enrichment analysis of upregulated DEGs; **(D)** Enrichment analysis of upregulated DEGs in KEGG pathway; **(E)** GO functional enrichment analysis of downregulated DEGs; **(F)** Enrichment analysis of KEGG pathway for downregulated DEGs.

In total, 1,059 DEGs were detected, including 401 upregulated and 658 downregulated genes (Figure 2A). The expression profiles of the DEGs were visualized via hierarchical clustering heatmap (Figure 2B).

GO enrichment analysis of DEGs (Figure 2C) showed that BP mainly involves mitotic nuclear division, sister chromatid segregation, mitotic spindle organization, etc; MF mainly involves microtubule binding, protein kinase regulator activity, ATP-dependent microtubule motor activity, plus-end-directed, etc; CC mainly involves kinetochore, mitotic spindle, etc. KEGG enrichment analysis of the DEGs (Figure 2D) showed that the main significantly enriched pathways included Cell cycle, Proteoglycans in cancer, and Estrogen signaling pathway. The GO enrichment analysis of DEGs (Figure 2E) showed that BP mainly involves fatty acid metabolic process, epithelial cell proliferation, response to hypoxia, etc; MF mainly involves organic acid binding, retinol dehydrogenase activity, oxidoreductase activity, etc; CC mainly involves collagen-containing ECM, intrinsic component of external side, cell-substrate junction, etc. KEGG enrichment analysis of the core targets (Figure 2F) showed that the main significantly enriched pathways included the *PPAR* signaling pathway, and the FoxO signaling pathway.

**FIGURE 3:**
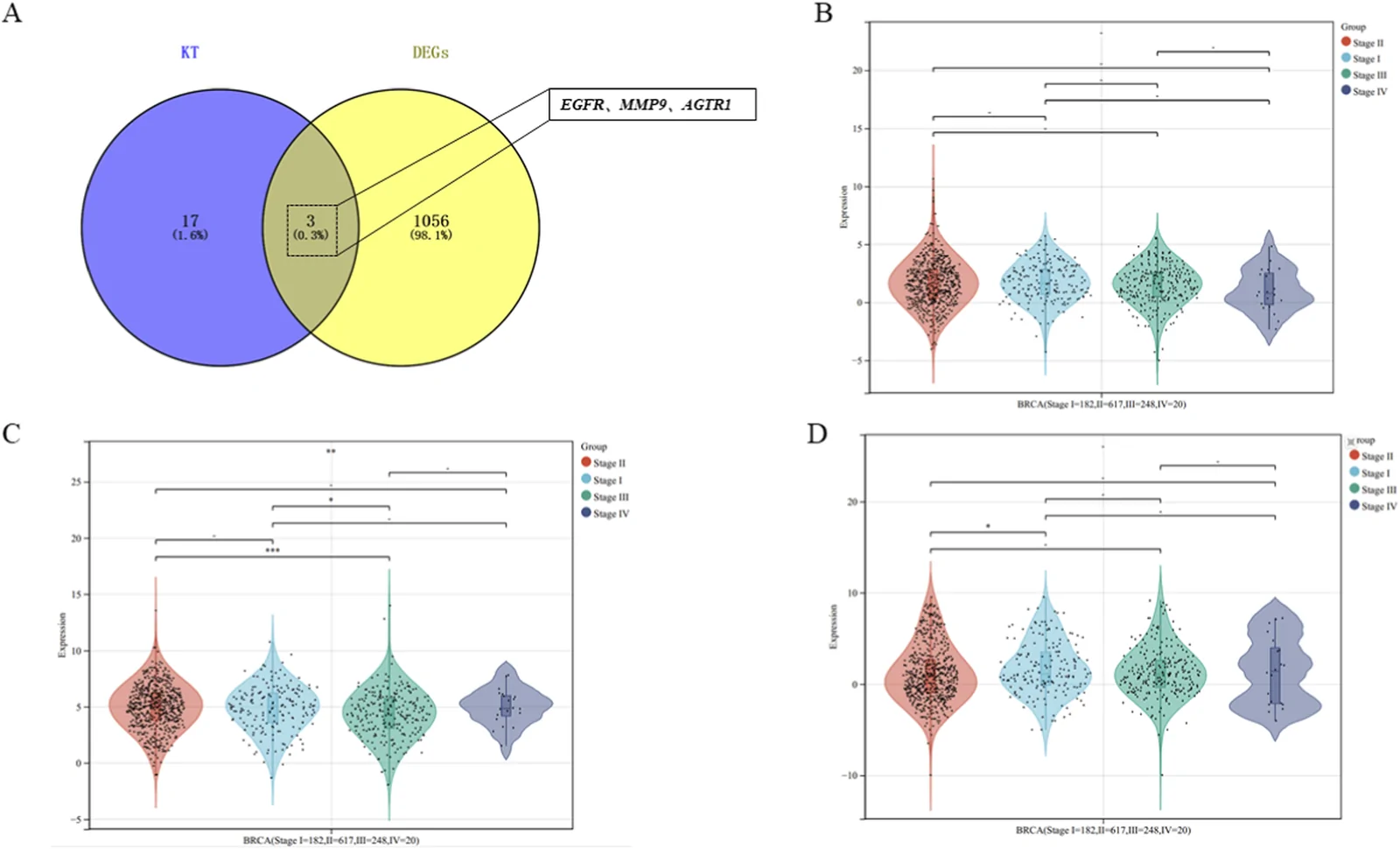
Screening of BC Hub genes and their expression in different clinical stages. **(A)** Hub gene screening; **(B–D)** The expression boxplots of *EGFR*, *MMP9*, and *AGTR1* in different clinical stages of BC.

Take the intersection between the top 20 key targets and DEGs. Three Hub genes, *EGFR*, *MMP9*, and *AGTR1*, were identified through screening (Figure 3A).

The expression results of the Hub gene in different clinical stages of BC revealed no statistically significant difference in *EGFR* (Figure 3B) and *AGTR1* (Figure 3D) expression levels among various stages, while the expression level of *MMP9* showed a statistically significant difference among the clinical stages, indicating that MMP9 may serve as a potential biomarker for BC progression (Figure 3C).

**FIGURE 4:**
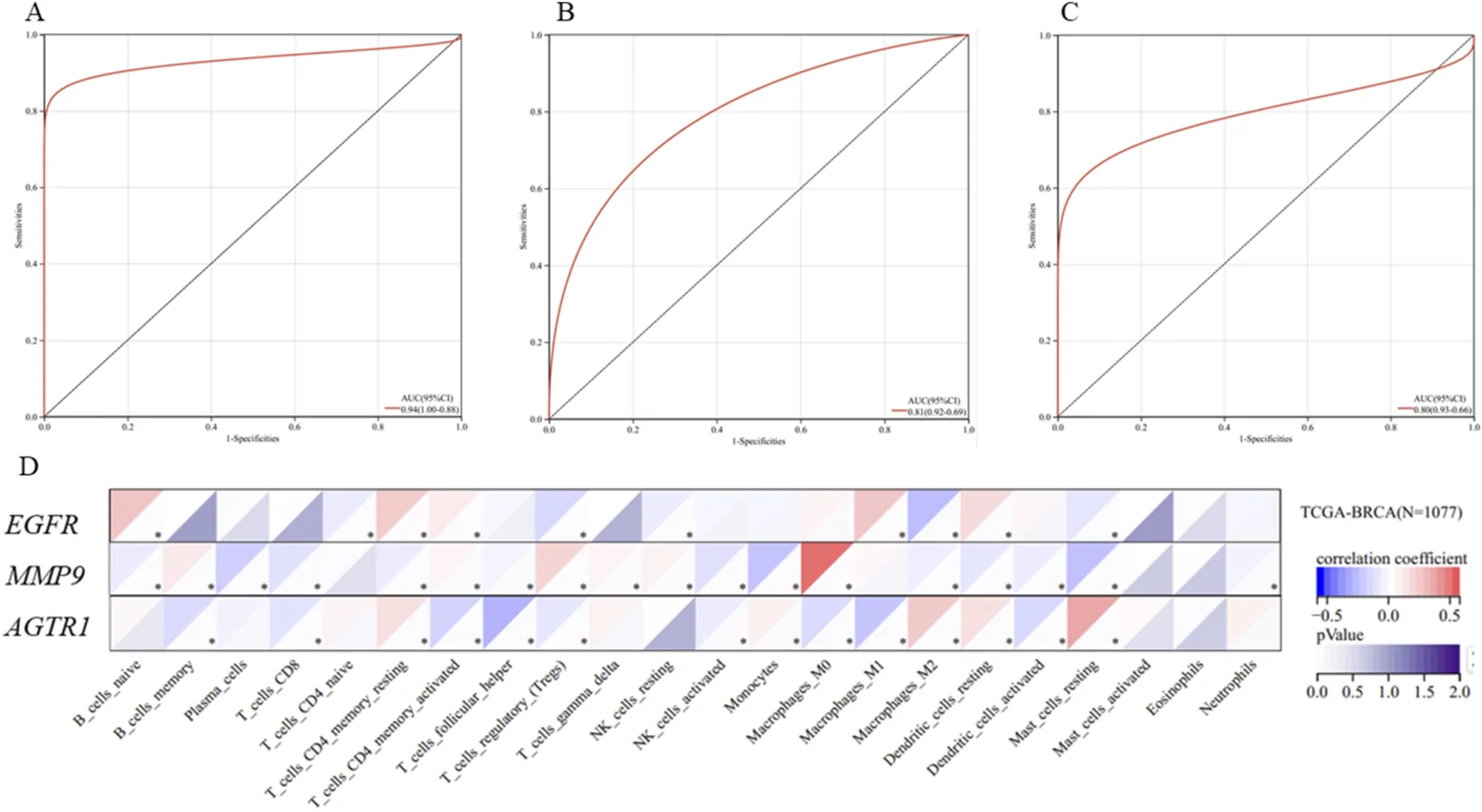
Diagnostic value and immune infiltration analysis of the Hub gene in BC. **(A–C)** ROC curves for the diagnostic efficacy of *EGFR*, *MMP9*, and *AGTR1* in BC, respectively; **(D)** Correlation heatmap between the expression of *EGFR*, *MMP9*, *AGTR1*, and the infiltration of 22 immune cell subpopulations in the TCGA-BRCA cohort.

The ROC curve analysis yielded AUC values of 0.94, 0.80, and 0.81 for EGFR, MMP9, and AGTR1, respectively. The closer the AUC is to 1, the higher the diagnostic value, indicating that *EGFR*, *MMP9*, and *AGTR1* have high diagnostic value for BC (Figures 4A–C).

To dissect the immunological relevance of *EGFR*, *MMP9*, and *AGTR1* in BC, we quantified the infiltration abundances of 22 immune cell subsets in the TCGA-BRCA cohort (n = 1,077) using the CIBERSORT deconvolution algorithm and analyzed their correlations with hub gene expression. Our results demonstrated that *EGFR* expression was significantly positively correlated with the infiltration levels of naive B_cells_naive, T_ cells_ CD4_ memory_ resting, Macrophages_ M1, and Dendritic_ cells_ resting B. In contrast, inverse correlations were observed between *EGFR* expression and the infiltration of T_ cells_ CD4_ naive, T_ cells_ regulatory_(Tregs), Macrophages_ M2, and Mast_ cells_ resting.

For *MMP9*, its expression showed positive associations with B_ cells_ memory, and Macrophages_ M0. Conversely, *MMP9* expression was negatively correlated with the infiltration of Plasma_ cells, Monocytes, and Mast_ cells_ resting.

Regarding *AGTR1*, its expression levels were positively associated with T_ cells_ CD4_ memory_ resting, Macrophages_ M2, and Mast_ cells_ resting, while exhibiting significant negative correlations with T_ cells_ CD4_ memory_ activated, T_ cells_ follicular_ helper, and Macrophages_ M1 (Figure 4D).

### 3.4 Subcellular localization of hub targets and its therapeutic significance

**FIGURE 5:**
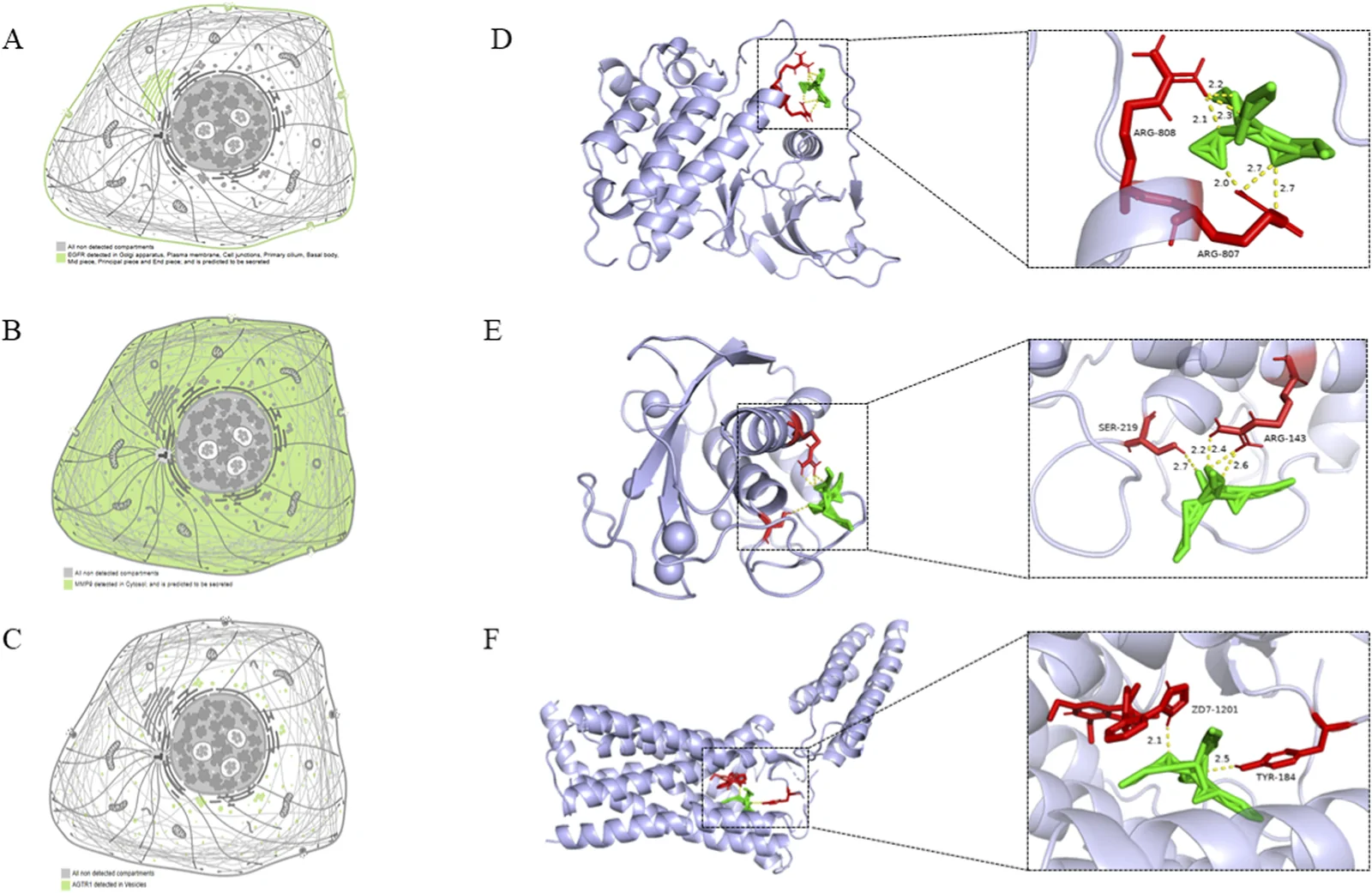
Hub gene localization, tissue expression validation, and docking with ATBC molecules. **(A–C)** Subcellular localization of *EGFR*, *MMP9*, and *AGTR1*, respectively; **(D–F)** Molecular docking of ATBC with *EGFR*, *MMP9*, and *AGTR1*, respectively.

Subcellular localization of the hub genes was analyzed using the THPA database. The results indicated that *EGFR* was mainly distributed in the cell membrane and Golgi apparatus (Figure 5A), *MMP9* was widely present in the cytoplasm (Figure 5B), and *AGTR1* was primarily localized to vesicles (Figure 5C).

These positioning patterns have clear mechanisms and therapeutic significance: *EGFR* is located on the cell membrane, confirming its surface receptor properties and can directly bind to the extracellular ligand ATBC, consistent with molecular docking results. At the same time, the distribution characteristics of this membrane also provide clinical theoretical basis for targeted intervention of *EGFR* with monoclonal antibodies and small molecule TKIs. The cytoplasmic localization of *MMP9* is consistent with its characteristic as a secreted protease: it is synthesized and processed in the cytoplasm and secreted to the extracellular matrix, degrading the basement membrane. This positioning provides a rationale for evaluating protein synthesis at the mRNA level in this study (He et al., 2023). The vesicle localization of *AGTR1* suggests that the G protein coupled receptor may rapidly internalize and recycle upon binding to ligands, thereby affecting the duration and intensity of ATBC induced signals (Karnik et al., 2015). These positioning data indicate that ATBC can directly bind to the above-mentioned targets and provide a theoretical basis for differentiated targeted interventions.

### 3.5 Molecular docking and visualization analysis

Molecular docking simulation was conducted to assess the direct binding affinities between ATBC and the three hub proteins (*EGFR*, *MMP9*, and *AGTR1*). A binding free energy threshold of < −5.0 kcal/mol was set as indicative of favorable binding affinity, while values < −7.0 kcal/mol indicated strong and stable binding activity. Detailed molecular docking energy data are summarized in Table 3. The results showed that the binding energies of ATBC with *EGFR*, *MMP9*, and *AGTR1* were all < −5.0 kcal/mol, suggesting that ATBC has good binding activity with the three hub target proteins. The molecular docking results were visualized (Figures 5D–F).

**TABLE 3:** Molecular docking results of hub gene and ATBC.

Key targets	Substance	PDB number	Binding energy (Kcal/mol)
*EGFR*	ATBC	1m14	−5.6
*MMP9*	ATBC	6esm	−5.6
*AGTR1*	ATBC	4YAY	−5.8

### 3.6 *In vitro* pharmacological assays

**FIGURE 6:**
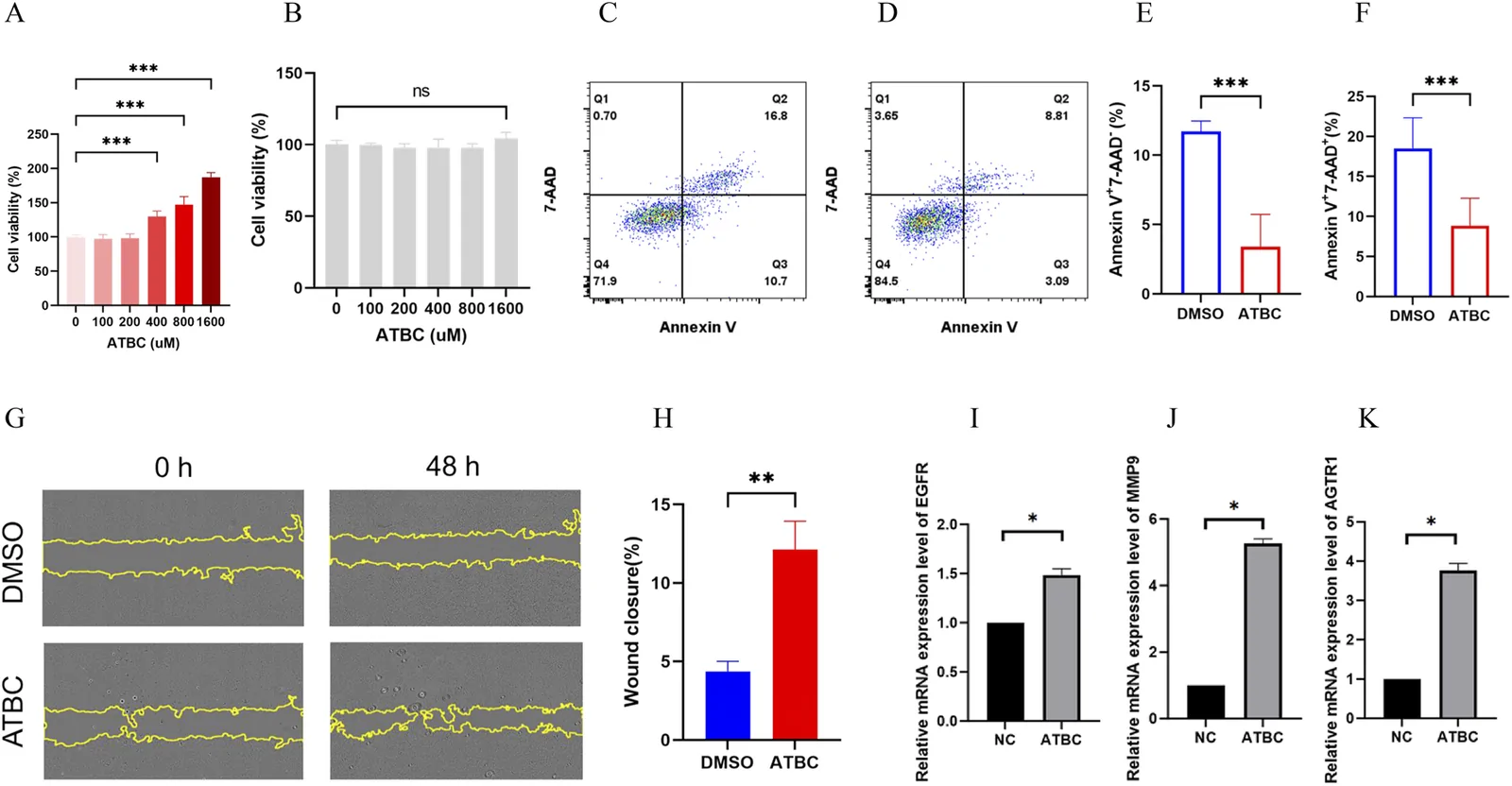
Effects of ATBC on the biological functions and mRNA expression of Hub genes in BC cells. **(A,B)** CCK-8 assay showing the effects of 24 h gradient concentrations of ATBC treatment on the viability of MCF-7 and MDA-MB-231 cells. **(C,D)** Flow cytometry detecting apoptosis in MCF-7 cells of the DMSO control group and 400 μM ATBC treatment group. **(E,F)** Quantitative analysis of the early apoptosis rate and late apoptosis/necrosis rate in MCF-7 cells. **(G)** Representative wound healing images of MCF-7 cells in the DMSO control and ATBC treatment groups at 0 h and 48 h. **(H)** Quantitative analysis of the wound healing rate between the two groups. **(I–K)** q RT-PCR results showing that ATBC significantly upregulates the mRNA expression of *EGFR*, *MMP9*, and *AGTR1* in MCF-7 cells.

Cell viability assays using the CCK-8 method demonstrated that ATBC exerted a concentration-dependent proliferative effect on MCF-7 cells, with 400 μM ATBC significantly enhancing cell proliferation compared with the 0 μM vehicle control (*P* < 0.001, Figure 6A). In contrast, ATBC concentrations up to 1,600 μM had no significant impact on the viability of triple-negative MDA-MB-231 cells (*P* > 0.05, Figure 6B). The CCK-8 cell viability assay showed that ATBC had a concentration dependent proliferative effect on MCF-7 cells: 400 µM ATBC significantly enhanced cell proliferation (compared to the 0 µM control group, *P* < 0.001, Figure 6A). When the concentration of ATBC reached 1,600 µM, it had no significant effect on the viability of triple negative MDA-MB-231 cells (*P* > 0.05, Figure 6B). This difference is likely related to the different molecular characteristics of the two: MCF-7 is ER positive, Luminal A subtype, and its proliferation is highly dependent on the estrogen signaling pathway. Previous studies have confirmed that ATBC has endocrine disrupting activity and can regulate the estrogen receptor pathway (Zhang D. et al., 2024; Lin et al., 2025). Therefore, the pro proliferative effect of ATBC in MCF-7 cells may be achieved through an ER dependent mechanism. On the contrary, MDA-MB-231 lacks functional ER signaling and is therefore insensitive to the endocrine disrupting effects of ATBC. These results suggest that ATBC exposure may preferentially promote the progression of hormone receptor positive BC subtypes.

Flow cytometric analysis of apoptosis revealed that ATBC treatment markedly reduced the total apoptotic rate of MCF-7 cells relative to the DMSO control group. Specifically, the early apoptotic rate was decreased by 3.43-fold (*P* < 0.001), and the late apoptosis/necrosis rate was reduced by 2.09-fold (*P* < 0.001, Figures 6C–F). Wound healing assays further demonstrated that 48-h treatment with 400 μM ATBC significantly accelerated the wound closure rate of MCF-7 cells (*P* < 0.001, Figures 6G,H). Corroborating our functional assay results, qRT-PCR analysis revealed that ATBC treatment significantly upregulated the mRNA transcript abundances of *EGFR*, *MMP9*, and *AGTR1* in MCF-7 cells relative to untreated controls (*P* < 0.05, Figures 6I–K).

## 4 Discussion

This study represents a target discovery phase investigation, integrating computational predictions with preliminary experimental validation to identify candidate molecular mediators of ATBC-driven BC progression. This study systematically investigated the molecular mechanisms by which ATBC exposure drives BC progression, employing a comprehensive Network Toxicology approach combined with multidimensional bioinformatics and *in vitro* experimental validation. Unlike traditional toxicological assessments, our study aimed to translate environmental exposure risks into specific interventions. By mapping the interaction network between ATBC and BC, we identified *EGFR*, *MMP9*, and *AGTR1* as core actionable targets. Our findings suggest that ATBC promotes BC progression by perturbing oxidative stress homeostasis, tumor-associated signaling pathways, and the tumor immune microenvironment. Crucially, identifying these targets provides a robust scientific basis for developing integrated targeted therapies against environmentally driven BC.

Initial computational predictions confirmed the potential carcinogenic risk of ATBC, consistent with previous toxicological reports indicating its role in inducing oral squamous cell carcinoma (Guo et al., 2025) and potential thyroid toxicity (Lan et al., 2025). Notably, emerging evidence also suggests that ATBC may possess endocrine-disrupting properties (Lin et al., 2025), which could be particularly relevant to hormone-dependent cancers such as BC. Building upon this, our systems pharmacology analysis isolated 58 potential targets of ATBC in BC. The top hub targets were significantly enriched in cell cycle phase transitions, cyclin-dependent protein kinase activities, and critical signaling cascades such as the FoxO pathway and proteoglycans in cancer. Dysregulation of these kinases and cell cycle regulators is a hallmark of BC proliferation and therapy resistance (Sun et al., 2023; Liu et al., 2022; Pola-Véliz et al., 2025; Zhou et al., 2025; Jiang et al., 2025; Zhang Y. et al., 2024). Proteoglycans act as critical mediators of pivotal signaling cascades such as TGF-β and Wnt/β-catenin, and exert dual modulatory functions in BC pathogenesis. This bidirectional regulatory activity positions proteoglycans as promising candidates for both prognostic stratification and precision targeted therapy of this malignancy (Vigo-Díaz et al., 2025; Quattrociocchi et al., 2025). The FoxO signaling pathway, in particular, acts as a crucial bidirectional regulator of tumor cell proliferation and epithelial-mesenchymal transition (EMT) (Dilmac et al., 2022; Contreras-Rodríguez et al., 2023). These results indicate that ATBC acts as an exogenous disruptor of intracellular kinase networks, offering a clear rationale for deploying specific kinase inhibitors as a targeted countermeasure.

By intersecting the key targets with BC-specific DEGs, we pinpointed three core hub genes: *EGFR*, *MMP9*, and *AGTR1*. Importantly, the three targets were significantly differentially expressed in BC. The identification of these specific cell surface receptors and intracellular/secreted mediators perfectly aligns with the development of targeted anti-cancer strategies. *EGFR* is a classic cell surface oncogenic driver. Its sustained activation drives cell proliferation, invasion, and EMT, significantly enhancing the distant metastatic potential of BC (Ren et al., 2026; Liu et al., 2023; Kang et al., 2024). Our *in vitro* data demonstrated that ATBC profoundly upregulates *EGFR* mRNA expression in MCF-7 cells, suggesting that environmental pollutants may confer a more aggressive phenotype. Consequently, targeting this cell surface receptor with existing *EGFR* tyrosine kinase inhibitors or monoclonal antibodies could serve as a highly effective intervention for ATBC-exposed BC patients. *MMP9* acts as a core intracellular and extracellular regulator that degrades the physical barrier of the ECM, promoting tumor invasion and angiogenesis (Yang et al., 2024; Tian et al., 2022). Its significant upregulation by ATBC highlights the potential of deploying MMP inhibitors to halt pollutant-accelerated metastasis. *MMP9* is located in the cytoplasm, which is consistent with its function as a secreted protease synthesized within the cell and subsequently released outside the cell. *AGTR1* is abnormally overexpressed in a subset of BC cases, particularly in the ER-positive/HER2-negative luminal subtype, and is strongly associated with treatment resistance and poor prognosis (Ma et al., 2019; Mei et al., 2024). *AGTR1* is located in vesicles, indicating that the dynamic transport process of receptors can regulate the duration and activation intensity of signals. The upregulation of this cell surface receptor under ATBC exposure broadens the potential for drug repurposing. Clinically approved Angiotensin Receptor Blockers could potentially be repurposed as integrated targeted therapies to block the ATBC-induced *AGTR1* signaling cascade, suppressing tumor progression.

This study found that BC cell lines showed a cell specific response to ATBC exposure: ATBC can significantly promote the proliferation of ER positive MCF-7 cells, but has no significant effect on triple negative MDA-MB-231 cells, suggesting that this effect may be mediated by endocrine mechanism. Previous studies have shown that ATBC has estrogenic activity and can regulate the ER signaling pathway (He et al., 2025; Xian et al., 2025). Therefore, we speculate that ATBC may act as an estrogen, bind to ER α in hormone receptor positive BC cells, trigger *EGFR* trans activation and downstream proliferation signal transduction, thus explaining its selective effect on MCF-7 cells expressing functional ER α, but not on MDA-MB-231 cells lacking ER. This cell specificity has important clinical significance, suggesting that ATBC exposure may be involved in the progression of luminal BC.

Furthermore, ROC curve analysis confirmed the high diagnostic value of these three genes, suggesting their utility as predictive biomarkers for pollutant-driven BC. Beyond their roles in tumor cells, the immune microenvironment exhibits significant heterogeneity that dictates patient prognosis and treatment response (Yan et al., 2024; Harris et al., 2024). To further elucidate the immunological relevance of the identified actionable targets, we conducted a systematic analysis of immune cell infiltration patterns in BC tissues. The results consistently showed that the expression of *EGFR*, *MMP9*, and *AGTR1* was tightly correlated with the infiltration densities of multiple functionally distinct immune cell subpopulations, with particularly pronounced associations observed for Tregs and M2-polarized macrophages. This strongly implies that ATBC exposure may foster an immunosuppressive microenvironment. From a perspective, targeting *EGFR*, *MMP9*, or *AGTR1* could not only directly inhibit tumor cell proliferation but also potentially reshape the tumor immune microenvironment, thereby sensitizing environmentally driven tumors to emerging immunotherapies.

This study has several limitations that should be acknowledgednoted. First, only the upregulation of hub gene mRNA expression by ATBC was verified, while protein-level validation and functional rescue experiments to establish clear causal relationships are lacking. Second, ur findings provide associative rather than definitive causal evidence. Functional rescue experiments and *in vivo* animal studies are required to establish causal relationships between ATBC exposure, target upregulation, and BC progression. These experiments are currently ongoing and will be reported in future work. Third, the bioinformatics analyses were based on public database mining and lacked external validation using independent BC clinical cohorts. Fourth, the ATBC concentrations used in the *in vitro* experiments differ from actual environmental exposure levels. Subsequent research should focus on the effects of long-term low-dose ATBC exposure, which is more consistent with the real exposure scenarios of the population.

In conclusion, this study bridges the gap between environmental toxicology and precision oncology by identifying *EGFR*, *MMP9*, and *AGTR1* as actionable targets in ATBC-driven BC. Our findings demonstrate that ATBC induces cell proliferation and migration while inhibiting apoptosis, particularly in ER-positive BC cells, mediated by the significant upregulation of these core genes. Cell line specific responses indicate potential endocrine disruption mechanisms that require further investigation. By mapping these interactions to specific cell surface and intracellular targets, we provide a compelling framework for future drug repurposing and the development of integrated targeted therapies to mitigate the oncogenic impact of environmental pollutants.

## Data Availability

All raw data can be requested from the corresponding author upon reasonable application.
